# Effectiveness of whole-body electromyostimulation on knee pain and physical function in knee osteoarthritis: a randomized controlled trial

**DOI:** 10.1038/s41598-024-71552-7

**Published:** 2024-09-06

**Authors:** Stephanie Kast, Wolfgang Kemmler, Frank W. Roemer, Matthias Kohl, Adam G. Culvenor, Ali Mobasheri, Michael Uder, Simon von Stengel

**Affiliations:** 1 Institute of Medical Physics, Friedrich-Alexander University Erlangen-Nürnberg, Henkestrasse 91, 91052 Erlangen, Germany; 2 Institute of Radiology, University Hospital Erlangen, Erlangen, Germany; 3 Department of Medical and Life Sciences, University of Furtwangen, Schwenningen, Germany; 4 Department of Radiology, Boston University Chobanian & Avedisian School of Medicine, Boston, MA USA; 5 La Trobe Sport and Exercise Medicine Research Centre, School of Allied Health, Human Services and Sport, La Trobe University, Bundoora, VIC Australia; 6 Oulu University, Oulu, Finland; 7 Department of Regenerative Medicine, State Research Institute Centre for Innovative Medicine, Vilnius, Lithuania

**Keywords:** Musculoskeletal system, Osteoarthritis, Chronic pain, Osteoarthritis, Randomized controlled trials, Pain management

## Abstract

In a randomized, controlled study, whole-body electromyostimulation (WB-EMS) was investigated as a promising alternative treatment technique compared to conventional strength training for the management of knee osteoarthritis (OA). Seventy-two overweight participants with symptomatic knee OA were randomly assigned to WB-EMS (n = 36) or a usual care group (UCG, n = 36). For seven months, the WB-EMS group received three times per fortnight a WB-EMS training, while the UCG was prescribed six-times physiotherapeutic treatments. We observed significant effects for the primary outcome “pain”, as determined by the Knee injury and Osteoarthritis Outcome Score (KOOS), with more favourable changes in the WB-EMS group vs UCG (between-group difference 9.0 points, 95%CI 2.9–15.1, *p* = 0.004). Secondary outcomes, including the other KOOS subscales (symptoms, function in daily living, function in sports/recreational activities and quality of life), 7 day pain diary, hip/leg extensor strength and lower limb function (30s sit-to-stand test), were also statistically significant in favour of the WB-EMS group. Overall, WB-EMS was found to be effective in relieving knee pain symptoms and improving physical function in individuals with symptomatic knee OA compared to usual care treatment. WB-EMS could be used as an alternative therapy in the management of knee OA; particularly for patients that cannot be motivated for conventional training.

## Introduction

Knee osteoarthritis (OA) is a leading cause of global disability^1^. The individual burden and socioeconomic impact of knee OA is profound and is expected to increase in the coming decades^2–4^. With no cure for OA currently, clinical guidelines emphasize treatments that relieve symptoms of the disease and improve function, such as exercise, weight loss (for those overweight) and education^5–7^.

Various exercise programs, such as resistance and endurance training, have a positive effect on pain and function in knee OA^8^. In a recent systematic review, resistance training was effective in reducing pain and/or improving function in daily living in 11 out of 12 studies (with a moderate to large effect size)^9^. In addition to local neuromuscular effects, systemic mechanisms that might modulate inflammatory processes are increasingly being discussed as mechanisms of action. Overweight and obesity might be negatively involved in this process and are a strong risk factor for the development and progression of knee OA^3,10,11^. Study results suggest that not only is the higher mechanical stress associated with obesity, but in particular visceral fat with its pro-inflammatory effect plays a role in the development and progression of OA groups^12,13^.

However, despite the high level of evidence regarding the benefits of physical activity and exercise for knee OA, the majority of individuals with knee OA do not meet recommendations for physical activity^14^. In individuals with knee OA, a vicious cycle of pain, avoidance of physical activity, reduced muscle strength and further functional limitations has been proposed^15^. As such, there can be barriers for participation in resistance training to improve strength^16^.

In contrast to conventional resistance exercise, Whole-body Electromyostimulation (WB-EMS) is a training approach characterized by intense activation of muscles with low voluntary effort. During WB-EMS training, all muscle groups of the trunk and extremities are simultaneously brought to contraction by electrical impulses using special suits with surface electrodes. WB-EMS technology simultaneously stimulates up to 14–18 regions or 8–12 muscle groups with up to 2800 cm^2^ electrode area, whereby the impulse intensity is separately adjustable for the different muscle groups. This approach may be an attractive alternative for individuals with knee OA who may have an inability to sufficiently voluntarily contract muscles to facilitate muscle strength gains and associated symptomatic relief. In previous studies, WB-EMS has shown positive effects on muscle strength, muscular morphology and fat mass in healthy, sarcopenic and/or functionally impaired participants^17–23^. Knee OA is associated with a lower proportion of total body muscle mass^24^ and increased comorbidities for diseases such as diabetes mellitus and cardiovascular disease and increased mortality due to reduced mobility and muscle activity^25^. Therefore, the whole-body training approach using WB-EMS could be particularly valuable for knee OA patients, as all the big muscles are activated, with positive systemic effects on fitness, total body composition, glucose metabolism and cardiovascular risk factors^26^ associated with inflammatory reactions. Anti-inflammatory effects, which are induced by the activation of large muscle groups, have been shown to be one potential mechanism of action of the health effects of WB-EMS^27^.

The aim of this study was to compare the effects of a 7 months WB-EMS application to a usual care control group (UCG) in overweight individuals with symptomatic knee OA. Our primary hypothesis was that WB-EMS will result in significantly greater reductions in knee pain compared to the UCG. We further hypothesized that, compared to the UCG, WB-EMS will result in significantly greater improvements in self-reported function in daily living, recreational activities and quality of life, hip/ leg extensor strength and physical function.

## Method

### Study design

The EMSOAT (Whole-Body Electromyostimulation for the Treatment of knee OA) study is a multi-center parallel-group (1:1 allocation) superiority randomized controlled trial (RCT) conducted at the Institute of Medical Physics (IMP), Friedrich-Alexander University of Erlangen-Nürnberg (FAU), and the Department of Radiology, University Hospital Erlangen Germany. The RCT was approved by the FAU ethics committee (Nr. 352_20 B) and all participants provided written informed consent prior to enrolment. The project fully complies with the Helsinki Declaration^28^ and was prospectively registered at clinicaltrials.gov, NCT05672264, on 05/01/2023.

### Participants

Participants were recruited between March and June 2022 in the metropolitan area of Erlangen-Nürnberg, Germany. As in previous studies, we recruited potential participants by reports and expert interviews on knee OA and corresponding study calls in local newspapers and social media. The call listed the key study eligibility criteria, contact person and an email address. Furthermore, we contacted eight medical practices (practitioners with qualification in sports medicine and orthopaedists) via letter and provided information flyers for their patients.

Inclusion criteria were (1) men or women 40–70 years of age, with (2) overweight (BMI > 25 kg/m^2^), (3) confirmed femorotibial OA equivalent to Kellgren-Lawrence grades (KL) 2 and 3^29^ (see explanation below), (4) knee pain for at least 3 months, (5) pain in the last 30 days at least on 50% of the days and (6) an average pain intensity > 2.5^30^ on a scale 0–10 (numeric rating scale (NRS)).

Exclusion criteria were: (1) Any WB-EMS training or more than 60min of resistance exercise training per week in the last year, (2) glucocorticoid or opioid medication in the last 3 months, (3) trauma to the knee joint or (4) intra-articular knee injection in the last 3 months, (5) conditions and diseases (and corresponding medication) with relevant impact on study outcomes (i.e. other rheumatic diseases e.g. rheumatoid arthritis, fibromyalgia, serious cardiovascular diseases), (6) conditions or diseases that are contraindications for WB-EMS (e.g. electric implants, epilepsy, cardiac pacemakers^31^) and (7) absence ≥ 4 weeks during the intervention period.

As radiographs could not be obtained for study purposes only^32^, potential participants were asked to provide externally acquired anterior–posterior radiographs of their index (more painful) knee when available. These were assessed by an experienced musculoskeletal radiologist (FWR) and those with KL 2 or KL3 were included^29^. Participants without externally acquired radiographs or radiographs older than 2 years were screened by MRI and those with full-thickness cartilage damage at both the femur and tibia in at least one compartment (grades 3.2 or 3.3 in at least one central femoral and one subregion of the anterior, central and posterior tibial subregions on the MOAKS (MRI Osteoarthritis Knee Score)^33^ scale) were excluded. Also, those with no or only focal cartilage damage (maximum of 1.0 or 1.1. in the 10 femorotibial subregions of the MOAKS instrument) were excluded. Using these MRI definitions, the likelihood of including KL 0 and 1 knees or knees with end stage structural OA (KL4) was minimized^34^.

If both knees of a single participant were eligible, we defined the side that caused more pain as the “index limb” (affected knee).

### Intervention

#### WB-EMS application

**Fig. 1:**
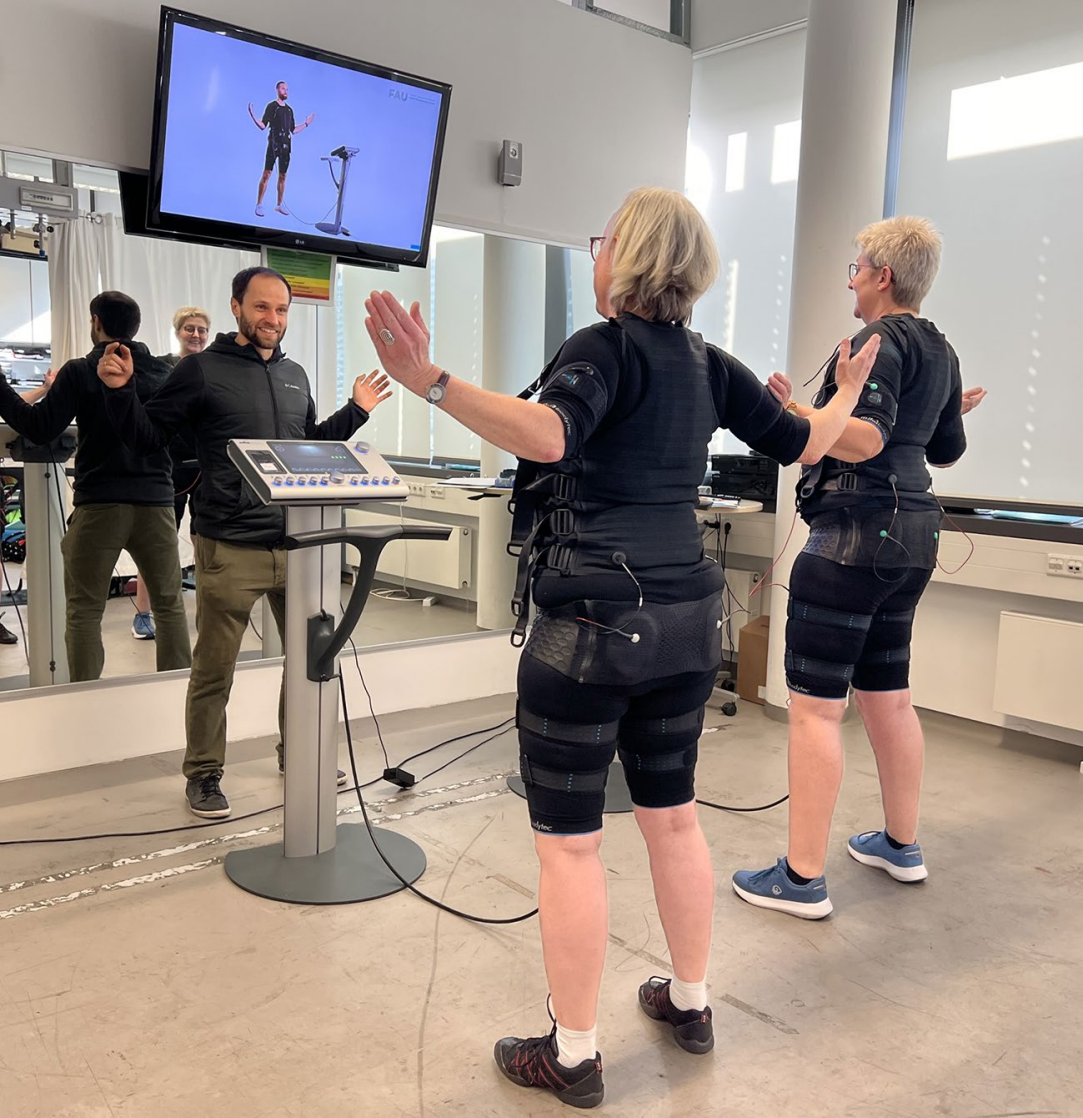
WB-EMS training session (Written informed consent was obtained from the participants to publish this picture)

WB-EMS was applied using a system with medical device approval (miha bodytec®, Type II, Gersthofen, Germany) that enables simultaneous stimulation of up to 10 main muscle groups (thighs and upper arms, hip/bottom, abdomen, chest, lower back, upper back, latissimus dorsi and two free options) with an overall area of stimulation of about 2800 cm^2^. The system allows intensities to be chosen for each region. We established a consistently supervised, video-guided WB-EMS program 3 times per fortnight (e.g. every Monday or Tuesday and every second Thursday or Friday) for 6 months (from August 2022 to January 2023) plus one month of conditioning (July 2022; see below). All participants started the intervention at the same time. We used an impulse protocol that was applied in research^18,19,21,22,35–37^ and most commercial settings in order to allow transferability of our approach. Bipolar electric current with a frequency of 85Hz, an impulse-width of 350 µs and a rectangular impulse pattern was used for 20 min in an interval approach with 6 s of EMS stimulation and 4 s of rest. Participants completed two sets with 6–8 repetitions of seven exercises (standing upright with trunk rotation, trunk flexion and extension, front abdominal press, diagonal side abdominal press; light dynamic squatting (with knee angles ≥ 120°) with arm chops, lateral pull; and light lunges with arm raises) in a standing position (Fig. 1). Of importance, we designed low-intensity movements/exercises to keep the effect of the voluntary movements itself as low as possible.

The intensity of the EMS was regulated based on the rate of perceived exertion (RPE) scale. We applied a perceived exertion rate to generate and maintain a sufficient but tolerable intensity of the EMS application. Before the 6 months of WB-EMS training, we implemented 4 weeks of conditioning with lower impulse intensity and shorter sessions (July 2022). We started with 12min in the first session and increased time by 2 min per session. After conditioning, participants were encouraged to exercise at an EMS-induced RPE of “6–7” (i.e. “hard + to very hard”) on the Borg CR10 Scale^38^. Impulse intensity was individually adapted for each body region in close interaction with the participant. During the session, instructors slightly increased (impulse) intensity every 2–3 min in close cooperation with the participants to maintain the prescribed RPE (“6–7”) during the session. From mid-September 2022, all participants used a second pair of circular electrodes for the thighs, to adequately stimulate the thighs and maintain the intensity. All training sessions took place in the Institute of Medical Physics. We applied a personal training setting with one licensed and experienced instructor responsible for two participants. Instructors monitored compliance with the prescribed exercise intensity and recorded attendance rate accurately. In case of non-participation, participants reported absence by email or telephone. Possible adverse events were recorded on a weekly basis during the entire course of the study. Further, the international guideline of safe and effective WB-EMS application was strictly respected^39^.

#### Control intervention

According to the German S2 guideline, physiotherapy measures in the form of strength and mobility training should be used for knee OA. In Germany, the attending physician prescribes 6 physiotherapy sessions as a standard treatment. In order to ensure that all participants in the UCG received this "usual care" standard procedure in the same way, all the UCG participants received a prescription for 6 physiotherapy treatment sessions (20 min each), which was prescribed by the study doctor and financed by study funds. The six treatment sessions should be carried out as a block at a rate of once a week within the first three months.

Three cooperating physiotherapy practices were recommended to carry out the therapy. However, participants were free to take the prescription to another practice of their choice. All practices were informed about the study and the aims of the study in a letter accompanying the prescription. The treatment contents were conducted individually in the sense of "usual care" in a diagnosis-orientated manner. This means that the specific content was decided by treating physiotherapists. Accordingly, techniques to reduce pain and to detonate the muscle tissue were used. Physiotherapists also worked on improving the mobility of the knee joint and strengthening the leg muscles depending on the individual findings.

Standardization was neither possible nor desirable, as the treatment was carried out by different practices and therapists. In addition, physiotherapy treatments on prescription are always individual and diagnosis-oriented, which is why we have chosen this procedure as "usual care".

#### Education (both groups)

Both groups were invited to participate in a training program for self-management of OA^40^. Six units (60min each) were offered over a period of 12 weeks. Before each of the 6 sessions, an invitation with a brief information was sent via email to the participants of both groups. The 6 sessions were led by different experts, each of them was blinded to the group allocation. The aim of the program was education, information and counselling to improve quality of life and mobility. Self-management, personal responsibility and coping strategies of the participants to cope with bio-psycho-social (stress) factors was promoted and supported. Overall, we intended to reduce fear and avoidance behaviour.

### Outcomes

Following outcomes were assessed at baseline and 7-months follow-up (FU):

**Primary outcome**

Pain subscale of the Knee injury and Osteoarthritis Outcome Score (KOOS-Pain)

**Secondary outcomes**

- The other four subscales of the KOOS questionnaire (see below)
- Knee pain intensity (7-day pain diary, NRS)
- Maximum strength of the hip/leg extensors (“leg press”)
- Objective lower-limb function (30 s sit-to-stand test)

**Exploratory outcomes**

- Total body-fat content and lean body mass
- Pain medication use (7-day pain diary)

#### Testing procedures

Participants were requested to refrain from intense physical activity and exercise 48 h before the assessments. Baseline and FU assessments were consistently performed by the same research assistant using the identically calibrated devices, in exactly the same setting and at about the same time of the day (± 90 min).

##### Anthropometry

Body mass and composition was determined through direct-segmental, multi-frequency Bio-Impedance-Analysis (DSM-BIA; InBody 770, Seoul, Korea). This device measures impedance of the trunk, arms and legs separately using an eight-point tactile electrode system that applies six frequencies between 1 and 1000 kHz.

##### Knee pain diary and questionnaire

Knee pain and self-reported functional status was determined using the KOOS questionnaire^41,42^ which comprises five subscales (dimensions): pain, other symptoms, activities of daily living (ADL), sports and recreation function (Sport/Rec) and knee-related quality of life (QoL). Each of these dimensions is scored separately, using a Likert scale with five possible answers ranging from 0 (no problems) to 4 (extreme problems). According to a formula, described in detail by Roos^41,42^, scores are transformed to a 0–100 scale, with zero representing extreme knee problems and 100 representing no knee problems.

In addition to the KOOS subscale pain, the intensity of knee pain was monitored using a numerical rating scale from 0 (no pain) to 10 (worst possible pain)^43,44^ conducted over 7 days, before and during the last week of the intervention. We provided standardized logs and requested the participants to rate their highest daily knee pain intensity every evening. The average 7 day pain intensity at baseline and FU was included in the analysis. Additionally, participants were asked to record pain medication daily in their logs. Average numbers of days using analgesics during the 7 day periods of monitoring were included in the analysis.

Lastly, we asked all participants in a baseline questionnaire for demographic parameters, diseases, medication and confounding lifestyle factors (physical activity, exercise and nutrition). The follow-up questionnaire specially addressed changes of this parameters in order to detect factors that may confound our results.

##### Functional testing

Maximum isokinetic hip-/leg-extension strength was tested using a linear isokinetic leg press (CON-TREX LP, Physiomed, Laipersdorf, Germany). Maximum strength was measured unilateral on the index limb (affected knee). Participants were sitting in a slightly supine (seatback 55°) position, fixed by hip and chest straps. Using the standard velocity of 0.5 m/s, range of motion was within 30° to 90° knee angle. After briefing and one familiarization trial with low effort, participants were requested to conduct two sets of five repetitions each with maximum voluntary effort (“push as strongly as possible”) separated by 60 s of rest. The highest force value of the two trials was included in the analysis. The present protocol has been applied in prior studies (e.g.^19,20,45,46^.).

In order to determine the physical function of the lower extremities (objective lower-limb function), the 30 s sit-to-stand test (“Chair Rise Test”) was used, which is a recommended performance-based test in individuals with knee OA^47^. With arms folded across their chests, participants were instructed to complete as many sit-to-stand movements as possible from a chair within 30s. Knees and hips had to be extended in the standing position, while the buttocks had to touch the seat in the lower position. Following a demonstration by the tester, a practice trial of one repetition was given to check proper form, followed by the 30 s test trial. We did not adjust the seat height for lower extremity length. The same standard chair was used for all assessments^48,49^.

### Sample size calculation

The sample size analysis was based on the primary endpoint of KOOS-Pain. Since there is a lack of data on the effect of WB-EMS in OA, the power analysis was based on the effects of conventional strength training on pain in knee OA. In the meta-analysis by Goh et al.^50^, a sub-analysis (89 studies; n = 7184) on the effect of strength training compared to "usual care" showed an SMD of 0.76 (0.50–1.02). With a power of 80% and an α-level of 5%, a two-sided t-test results in a required number of cases of n = 31/group. Since the meta-analysis of Goh et al. included predominately passive control groups, while our study implemented a usual care control group (6 physiotherapeutic sessions), we designed our sample size analysis more conservatively by increasing the number of cases by 15% which is equivalent to assuming an SMD of 0.67. Correspondingly, we aimed to include 36 subjects per group (WB-EMS: n = 36, UCG: n = 36).

### Randomization and blinding

Using two strata for pain intensity (NRS, assessed as inclusion criteria), the 72 eligible participants were allocated to the study groups based on drawing small opaque capsules placed in a bowl. In detail, 72 capsules were put in a bowl, prepared by a researcher not involved in the trial. Thus, neither participants nor researcher knew the allocation beforehand (allocation concealment). After the randomization procedure, the principal investigator (SK) registered participants and instructed them in detail about study specifications.

Our blinding strategy was applied for persons who assessed the outcome parameters, acquired and analysed data and thus, were kept unaware of the participants’ group status (WB-EMS or UCG) and were not allowed to ask, either.

### Statistical analysis

Intention to treat (ITT) analyses were applied. Multiple imputation (ITT) was performed using R statistics software (R Development Core Team Vienna, Austria^51^) in combination with Amelia II^52^. We used the full data set for multiple imputations, with imputation repeated 100 times. Over imputation diagnostic plots (“observed versus imputed values”) were checked by Amelia II. For pooling, the results R package mice^53^ was used. Additionally, we applied per protocol (PP) analyses for all participants with complete datasets (baseline and 7-months assessment), independent of their compliance, for all the primary and secondary study outcomes. The results of PP and ITT analyses were similar and identical with respect to significances. Assumptions, such as normal distribution, were checked graphically (qq-plots, residual plots). The changes over time within groups were analysed by paired t-tests. The group differences at follow-up (”effects “) were determined by ANCOVA, adjusting for baseline data using the group as covariate. Categorical variables were addressed using the Chi-Square test. Differences in use of pain medication (yes vs no) were determined by a two-way Analysis of Deviance (logistic regression) using the likelihood-ratio-test. All tests were 2-tailed and significance accepted at *p* < 0.05. According to the suggestion of Li et al.^54^, we did not adjust secondary outcomes for multiplicity. Standardized Mean Difference (SMD) according to Cohen (Cohen’s d)^55^ was also calculated to indicate the size of the effect for primary and secondary outcome variables. SMDs ≥ 0.2, 0.5 and 0.8 represent small, medium and large effect sizes.

## Results

**Fig. 2:**
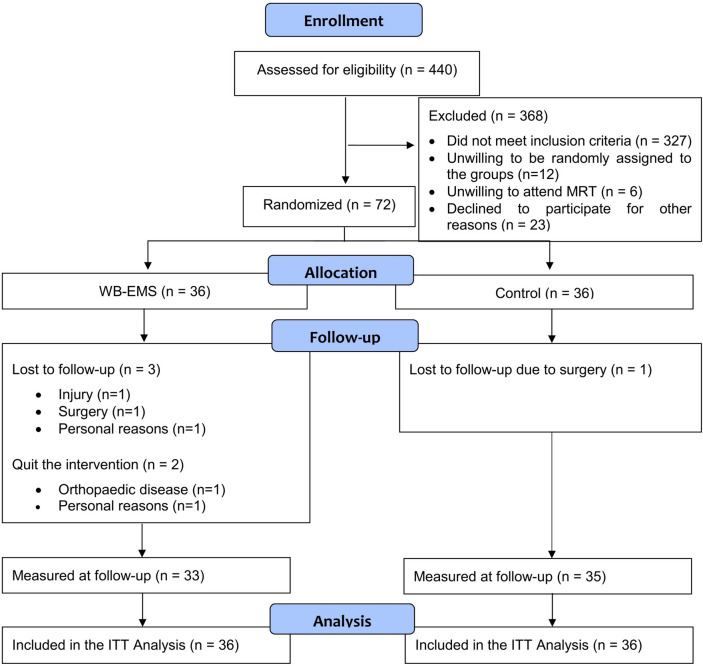
Study flow diagram (according to CONSORT, Consolidated Standards of Reporting Trial).

A total of 440 women and men responded by email or telephone. After sending detailed study information via email, potential participants were further assessed for eligibility by phone calls. Of the remaining 113 participants, 12 were unwilling to be randomly assigned to the groups, 6 were unwilling to attend MRI and 23 declined to participate for other reasons. Finally, 72 participants could be included in the study. Participant flow through the study is displayed in Fig. 2.

Table 1 lists the baseline data. No significant differences between the groups were observed for baseline values. Of the 72 subjects randomized, 4 subjects were lost to FU for reasons unrelated to the study (UCG: n = 1; WB-EMS: n = 3) (Fig. 2). Two participants of the WB-EMS group quit the intervention. One of these persons quit the trial after 11 weeks of training because of orthopaedic problems unrelated to the exercise program. The second person quit after 5 months of training because of personal reasons.

**Table 1:** Baseline characteristics of the study participants.

Variable	UCG (n = 36)	WB-EMS (n = 36)
Age (years)	57.9 ± 7.0	58.3 ± 7.2
Gender (women/men) [n]	24 / 12	22/14
Body mass index (BMI) [kg/m^2^]	29.3 ± 3.6	31.1 ± 4.6
Body height [cm]	174.3 ± 9.0	173.2 ± 9.9
Body mass [kg]	89.5 ± 15.1	93.2 ± 15.1
Lean body mass (LBM) [kg]	58.1 ± 11.8	60.2 ± 12.5
Total body fat [%]	35.0 ± 7.7	35.2 ± 9.2
Physical activity [Score]^1^	3.70 ± 1.11	3.58 ± 1.28
No exercise [n] ^2^	12 (33%)	13 (36%)
Knee pain intensity [NRS] ^3^	4.07 ± 1.61	4.05 ± 1.45

All values are expressed as mean value ± standard deviation.

UCG, usual care group; NRS, numeric rating scale (0–10); WB-EMS, whole-body electromyostimulation group.

^1^Self-rated physical activity (“very low” (1) to “very high” (7), assessed by questionnaire.

^2^Assessed by questionnaire.

^3^Average knee pain intensity, assessed by 7 day protocol.

On average, participants attended 88% ± 10% of WB-EMS sessions (3 times per fortnight) over the period of 7 months (including condition). In most cases, the reason given for the absence was illness, whereby three participants had longer periods (4–8 weeks) of inactivity due to viral infections. No adverse or unintended effects or injuries were observed during the WB-EMS sessions, and no participant reported any WB-EMS-related discomfort during or after WB-EMS application. More than 90% of the participants in the UCG have redeemed the prescription with the 6 physiotherapy treatments. The participation rate regarding the self-management program was around 50%. Both groups participated equally.

Table 2 displays the results of primary and secondary outcomes. KOOS-Pain scores improved significantly more in the WB-EMS group compared with the UCG (18.2% difference, mean absolute difference (MD) 9.0 points, 95% CI 2.9–15.1, *p* = 0.004). In Detail, the score improved by 12.5% within the UCG (*p* = 0.003) and by 30.7% within the WB-EMS (*p* < 0.001).

**Table 2:** Baseline data and changes of primary and secondary outcomes in the WB-EMS and UCG.

	UCG (n = 36) MV ± SD	WB-EMS (n = 36) MV ± SD	Difference MV (95% CI)	SMD d^1^	*p* value
KOOS Pain					
Baseline	56.1 ± 12.9	54.4 ± 12.4			
FU	63.1 ± 15.1	71.1 ± 13.9			
Changes	7.0 ± 13.6**	16.7 ± 13.9***	9.0 (2.9–15.1)	0.65	.004
KOOS Symptoms					
Baseline	57.5 ± 15.4	57.7 ± 14.5			
FU	61.7 ± 15.3	70.3 ± 13.4			
Changes	4.1 ± 13.8 ns	12.6 ± 14.1***	8.6 (2.8–14.4)	0.62	.004
KOOS ADL					
Baseline	64.6 ± 13.6	65.1 ± 13.9			
FU	68.0 ± 13.2	79.1 ± 12.6			
Changes	3.4 ± 13.7 ns	14.0 ± 13.9***	10.8 (5.3–16.3)	0.78	< .001
KOOS Sports/REC					
Baseline	33.1 ± 21.1	28.8 ± 20.8			
FU	41.4 ± 22.5	50.2 ± 19.2			
Changes	8.3 ± 18.7*	21.4 ± 19.1***	11.5 (3.3–19.6)	0.61	.007
KOOS QoL					
Baseline	33.3 ± 16.5	31.4 ± 13.2			
FU	39.1 ± 18.5	47.4 ± 13.6			
Changes	5.7 ± 14.3*	16.0 ± 14.7***	9.5 (3.1–16.0)	0.66	.004
Knee pain intensity (NRS)					
Baseline	4.07 ± 1.60	4.05 ± 1.45			
FU	3.31 ± 1.87	2.26 ± 1.29			
Changes	− 0.76 ± 1.73*	− 1.78 ± 1.75***	− 1.04 (− 1.75 to − 0.33)	0.60	.005
Maximum isokinetic Hip/Leg Extensor Strength [N]^2^					
Baseline	749.2 ± 224.8	798.5 ± 230.5			
FU	778.5 ± 235.6	903.4 ± 278.9			
Changes	29.3 ± 151.3 ns	104.9 ± 152.6***	79.0 (6.9–151.2)	0.52	.03
Sit-to-stand test (Chair Rise) [n]					
Baseline	17.7 ± 6.6	18.7 ± 5.9			
FU	18.2 ± 7.53	23.0 ± 5.74			
Changes	0.53 ± 4.06 ns	4.30 ± 4.07***	3.9 (2.0–5.8)	0.96	< .001

All values are expressed as mean value (MV) ± standard deviation (SD).

UCG, usual care group; CI, confidence interval; FU, 7-months follow-up; KOOS, Knee injury and Osteoarthritis Outcome Score (0–100, 0 = extreme problems, 100 = no problems); NRS, numeric rating scale (0–10, 0 = no pain, 10 = worst possible pain); SMD, standardized mean difference; WB-EMS, whole-body electromyostimulation group.

^1^d ≥ 0.2 small effect; d ≥ 0.5: moderate effect; d ≥ 0.8: high effect.

^2^measured unilateral (knee of interest).

*p < 0.05; ** *p* < 0.01; *** *p* < 0.001; ^**ns**^ non-significant (changes within groups).

All secondary outcomes (other KOOS subscales, NRS, sit-to-stand test, muscle strength) also improved significantly more in the WB-EMS group compared to the control group at 7 month FU (Table 2). More in detail, in KOOS-Symptoms score there was a net benefit in favour of the WB-EMS group of 14,7% (MD 8.6 points, 95% CI 2.8–14.4). The result for KOOS-ADL score was similar: WB-EMS improved the score by 16.2% compared to UCG (MD 10.8 points, 95% CI 5.3–16.3). The fourth and fifth KOOS dimensions Sport/REC and QoL also changed more favourably in the WB-EMS. The Sport/REC score was 49.2% (MD 11.5 points, 95% CI 3.3–19.6) and the QoL score was 33.9% (MD 9.5 points, 95% CI 3.1–16.0) higher in the WB-EMS group than in the UCG.

In parallel, the average knee pain intensity (NRS), which was recorded via 7 day diary, decreased significantly in WB-EMS by 25.3% compared to the UCG (MD -1.04, 95% CI − 1.75 to − 0.33). The number of “sit-to-stands” in 30s (Chair Rise) developed in favour of the WB-EMS compared to the UCG (MD 3.9 reps, 95% CI 2.0–5.8). In line with the changes in the sit-to-stand test, there was a significant between-group difference for change in maximum isokinetic hip/leg extensor strength (MD 79.0 N, 95% CI 6.9–151.2) favouring WB-EMS group.

Table 3 displays the results of the exploratory outcomes. In contrast to the results described above, the WB-EMS program did not lead to a significant change or between-group difference in body weight. With respect to body composition, lean body mass remained stable in WB-EMS, whereas it significantly decreased (*p* = 0.02) in the UCG. The difference between the groups was non-significant (*p* = 0.09). UCG significantly gained fat mass (Tab. 3), whereas the increase in fat mas in WB-EMS group was not significant. Again, the between group difference were not significant (Tab. 3).

**Table 3:** Baseline data and changes of exploratory outcomes in the WB-EMS and UCG.

	UCG (n = 36) MV ± SD	WB-EMS (n = 36) MV ± SD	Difference MV (95% CI)	SMD d^1^	*p* value
Body fat content [%]					
Baseline	35.0 ± 7.7	35.2 ± 9.2			
FU	36.2 ± 8.1	35.6 ± 9.1			
Changes	1.21 ± 1.95***	0.42 ± 2.02 ns	− 0.79 (− 1.73 to 0.15)	0.40	.098
Lean body mass [kg]					
Baseline	58.1 ± 11.8	60.2 ± 12.5			
FU	57.4 ± 11.7	60.1 ± 11.8			
Changes	-0.62 ± 1.58*	-0.08 ± 1.62 ns	0.62 (− 0.10 to 1.35)	0.39	.09
Pain medication [weekly dose]					
Baseline	0.81 ± 2.47	0.64 ± 1.33			
FU	1.36 ± 2.85	0.32 ± 1.40			
Changes	0.56 ± 2.38^ ns^	-0.31 ± 2.43 ns	− 0.98 (− 1.97 to 0.04)	0.41	.059

All values are expressed as mean value (MV) ± standard deviation (SD).

UCG, usual care group; CI, confidence interval; FU, 7-months follow-up; SMD, standardized mean difference; WB-EMS, whole-body electromyostimulation group.

^1^d ≥ 0.2 small effect; d ≥ 0.5: moderate effect; d ≥ 0.8: high effect.

* *p* < 0.05; ** *p* < 0.01; *** *p* < 0.001; ^**ns**^ non-significant (changes within groups).

No significant between-group differences with respect to physical activity (*p* = 0.106), exercise or diet were reported. The weekly intake of analgesics, assessed via 7 day protocol, tendentially increased in the UCG (BL: 0.81 ± 2.47; FU: 1.36 ± 2.85) and decreased in the WB-EMS (BL: 0.64 ± 1.33; FU: 0.32 ± 1.36). The intergroup difference was borderline non-significant (*p* = 0.059). Of note, the number of subjects who took oral analgesics, as determined via the 7 day protocol, was 8 in UCG and 9 in WB-EMS at baseline. At FU 10 participants in UCG and 2 in WB-EMS used oral analgesics. After 7 month of intervention a significant reduction of number of participants taking analgesics in the WB-EMS compared to UCG was observed (*p* = 0.033).

## Discussion

Our findings demonstrated that WB-EMS was highly effective in alleviating knee pain (KOOS Pain: + 18.2%, SMD 0.65, *p* = 0.004) and improving function of the knee (KOOS SMD 0.62–0.78) compared to a usual care group.

To our best knowledge, only one other study has evaluated the effect of WB-EMS in individuals with knee OA^27^. Park et al.^27^ examined the effectiveness of isometric strength exercise superimposed by WB-EMS (ISOM + WB-EMS) compared to isometric exercises alone or a non-training control. As in our study, knee pain and function were recorded using the KOOS questionnaire. It should be noted that the isometric exercises alone showed an effect on the KOOS scores symptoms, ADL, Sports/Rec and QoL compared to passive control (all *p* < 0.002). However, the WB-EMS application led to additional effects. The KOOS scores for pain, symptoms and ADL were significant higher in the ISOM + WB-EMS group compared to the exercise group alone (all *p* < 0.001)^27^.

However, as the pilot study of Park follows a fundamentally different training approach (strength exercises superimposed by WB-EMS), the results are hardly comparable. Unlike Park et al., we pursued a low-threshold approach in which the muscles are activated predominantly via EMS while performing light and less strenuous voluntary movements. This method might be attractive especially for the large target group of people who are not motivated or able (e.g. because of pain) to perform intensive and strenuous strength training exercises. Following our philosophy of low barriers, the training frequency was 3 sessions per fortnight, compared to 3 sessions per week in Park’s study. Further, with regard to the study population, Park et al. included individuals with early knee OA (KL 1–2) while knee pain was not an eligibility criterion. Accordingly, the baseline pain level was lower compared to our study (KOOS pain score was on average 18 points higher).

There are some studies on the effect of local EMS in knee OA in which the electrical current was only applied to the leg area (the term "neuromuscular electrical stimulation" (NMES) is usually used in the literature). The results of two recent meta-analysis on the effect of local EMS in individuals with knee OA did not show a significant reduction in pain using^56,57^. In the majority of local EMS studies, the quadriceps muscle was stimulated in isolation with adhesive electrodes. This approach appears suboptimal, considering the importance of the hamstring muscles and intermuscular and proprioceptive coordination for the stability of the knee joint^58^. Strengthening the hamstring muscles in addition to strengthening the quadriceps muscles has even been shown to be beneficial for pain symptoms, mobility and function in knee OA^59^. In WB-EMS, agonists and antagonists (e.g. quadriceps and hamstrings) are activated simultaneously over a large area by using cuff electrodes.

A recent study showed that WB-EMS has a positive effect not only on osteoarthritic knee pain, but also on lower back pain^60^. Average daily LBP intensity, as assessed by a pain dairy using NRS, changed more favourably in the WB-EMS group, compared with a control group (MD 0.67, 95% CI 0.18–1.24, SMD 0.75)^36^. The pain-relieving effect of WB-EMS could take place via different pathways. One pathway could be an improvement in knee joint stability through an increase in muscle strength, as we observed in our study. In addition, the electrical current itself, which is technically an identical form of current as used in the high-frequency transcutaneous electrical nerve stimulation (HF-TENS) method for pain reduction, could have contributed to the effect. In a recent meta-analysis, TENS currents proved to be effective for pain relief in knee OA^60^. The pain reducing effects of TENS is probably based on an activation of a complex neuronal network (gate control theory) and a release of endogenous opioids^61^. In comparison to the TENS method, which usually uses a light electric current (below motoric threshold), the stimulation intensity is higher in EMS. Finally, study results suggest that muscle activity is associated with the secretion of anti-inflammatory substances, which might be another mechanism of pain reducing effects of EMS^13,62^. There is some evidence of positive effects of WB-EMS application on inflammatory biomarkers in elderly women with early knee OA^27^. As mentioned in the beginning, laboratory parameters including inflammation markers will be published separately, as our trial is separated into different work packages.

Comparing our study results on the effect of WB-EMS on osteoarthritic knee pain with conventional strength training, a meta-analysis by Goh et al.^50^ shows that conventional strength training has a comparable effect with an SMD of 0.76. Conventional training also resulted in an improvement in function with an SMD of 0.78, comparable to our effects (KOOS ADL: SMD 0.78).

In the present study we observed a significant increase in maximum hip-/leg extension strength of 9%, when comparing the effect of WB-EMS with usual care. Strength is highly relevant for the function of the knee joint and reduced strength is also a risk factor for worsening OA^63^. For this reason, these results are very relevant, even if the increase in strength is only moderate. It should be noted that the results of the strength measurement on the isokinetic leg press represent the sum of the strength of the knee and hip extensor muscles in a functional way of multi-joint testing. In other studies with knee OA collectives, the strength of the quadriceps and hamstrings is usually measured on an isokinetic leg curler, which measures the force of the knee extensor or flexor muscles in the open kinetic chain in a single-joint isolated manner.

In Park's study, the development of the quadriceps muscle strength, measured as peak torque on a isokinetic dynamometer, was superior in the combined group that received superimposed WB-EMS compared to isometric exercises alone (+ 10%). This demonstrates an effect of electrical stimulation per se^27^.

Comparing the results with studies in which WB-EMS training was used in other cohorts show similar results. One study summarizes the increases in leg extension strength for different male age cohorts following WB-EMS training, using the same measurement technology on the isokinetic leg press. A tendency towards a decreasing effect of lower extremity strength gains with increasing age (35–49 years: 13.8 ± 9.8% change, 50–64 years: 11.7 ± 9.2, 65–79 years: 9.1 ± 7.3) was found^64^. Another study directly compared the effect of WB-EMS (1,5x/w 20 min) training with that of HIT strength training (2x/w 30 min), with no significant difference between the groups in terms of leg strength gains (p = 0.215), as determined using isokinetic leg-press (HIT 12.7 ± 14.7%, *p* = 0.002, versus WB-EMS 7.3 ± 10.3%, *p* = 0.012)^19^.

With regard to conventional strength training in knee OA, both high-intensity resistance training (RT) (70–80% of 1-repetition maximum) and low-intensity RT (40–50% 1-RM) led to an increase in knee extension and flexion peak torque^65^. The high-intensity training increased knee extension strength by 11.2% (flexion 9.8%), the low-intensity program 9.1% (8.9%). It should be noted that the training frequency was 3 training sessions per week.

The maximum strength determined on the leg press proved to be a more relevant measure of physical function (sit-to-stand, get up and go, stair climb) than the isolated quadriceps strength in the cohort of knee OA patients^66^. After the leg strength had improved, an improvement in the sit-to-stand test was also to be expected, which was confirmed by a significant difference between the groups in favour of the WB-EMS group (SMD of 0.96, *p* < 0.001).

Our WB-EMS program did not result in any significant intergroup differences in weight, muscle mass and fat mass, even though an increase in fat mass and a decrease in LBM was recorded within the UCG. A recent meta-analysis of Kemmler et al.^67^ shows significant effects of WB-EMS on muscle mass, but not on body fat mass. We cannot explain why an almost identical WB-EMS training showed a lower effect in this study. With respect to fat mass, the training volume of the time-efficient WB-EMS approach which requires only 30 min of training per week, is probably not sufficient to induce relevant changes of body composition.

Our project has various strengths. Great emphasis was placed on the safety aspect. This refers to an individual dosage and a slow progressive increase in intensity to ensure safety and adaptation of the muscles. To achieve that, we conducted 1 month of conditioning with an initial lower intensity (i.e. current intensity) and a shorter application duration to prepare the participants well for the WB-EMS training. The aim of this method was to avoid high levels of creatine kinase (CK) after initial applications^68^. Moreover, we wanted to ensure that the training sessions set over threshold stimuli for the whole period of 6 months. After the initial phase, an RPE target of “6–7” on the Borg CR10 was used. Lastly, the training was carried out by qualified trainers with a supervision ratio of 1:2 (trainer:participant) to ensure a high level of safety through optimal assistance and monitoring.

We observed a high attendance rate in our WB-EMS group (88%). This indicates that our exercise protocol was not only effective but obviously attractive, even in this cohort with a low affinity to conventional resistance training. The high attractiveness was confirmed by the low drop-out rate, as there were only 3 dropouts in the WB-EMS group (all were unrelated to the program). No participant showed intolerance to electrical stimulation and no EMS related side effects were reported.

Apart from its effectiveness and safety, high importance was attached to generalizability and transferability. We focused on overweight people who represent a large proportion of individuals with knee OA, because overweight is a strong risk factor for the development and progression of knee OA^3,10,11^. The pathogenetic processes are not only based on increased mechanical stress, but in particular on inflammatory reactions associated with increased visceral fat^12,13^. However, the transferability to lean cohorts should be investigated in further studies.

We applied a WB-EMS protocol and equipment used in the majority of the about 2,000 commercial settings alone in Germany^69^. This ensures a good transferability of the results and enables the findings to be applied more broadly using existing structures of commercial providers. Thus, the aspect of limited applicability cannot be considered as a barrier to start WB-EMS. Nevertheless, the application of WB-EMS in the medical setting is still limited, particularly in physiotherapy practices. This might be due to the presently limited billing capability by health insurance companies. Considering the personal training approach of WB-EMS and the corresponding expenses, the financial aspect might be the most striking factor against the widespread application of WB-EMS in therapy.

In order to rule out the possibility of the use of pain medication distorting the results, we recorded the medications as part of the pain diary. It was notable that the number of participants taking pain medication significantly decreased in the WB-EMS group and the amount of medication taken decreased tendentially, which excludes the possibility that the medication distorted the study results.

Some limitations of our trial should also be noted. One limitation is that it was not blinded at participant and investigator level. To be blinded at participant level, the UCG would have had to receive the identical intervention as the training group, with the difference that the WB-EMS devices would have provided electrical stimuli only below motorical threshold. However, since low-threshold electrical stimuli, applied as transcutaneous electrical nerve stimulation (TENS), showed pain-relieving effects in individuals with knee OA^60^, we did not use a blinded study design with low-intensity TENS, but pragmatically implemented a usual care group. In this context, it should be mentioned once again that the exercises performed during WB-EMS were designed in such a way that they should not lead to muscular adaptations. However, it cannot be ruled out that the dynamic movements without electrical stimulation also had a pain-relieving effect. Our design does not allow us to separate the possible effects of WB-EMS and the movements. Furthermore, it cannot be excluded that participation in the WB-EMS group led to a placebo effect, which could have influenced self-rated parameters in particular. Another limitation is that OA was not uniformly defined radiologically as an inclusion criterion using the Kellgren-Lawrence score. Since, for reasons of time and economy, no application was made to the Federal Office for Radiation Protection for the production of X-ray images, we examined existing X-ray images and, if not available or too old, MRI images were taken. However, with this procedure, the likelihood of including KL 0 and 1 knees or knees with end stage structural OA (KL4) was minimized^34^.

According to various international guidelines^6,7,70^, targeted physical training is a critical component of the treatment of knee OA. WB-EMS is a training approach that stimulates all the main muscle groups simultaneously, but each with dedicated intensity, and is known to have a wide range of positive health effects beyond knee OA^26^. In summary, we demonstrated that 3 times per fortnight of WB-EMS positively effects knee pain and function in individuals with knee OA. Studies in which conventional strength training was used report similar positive effects as in the present study, but are less time-efficient^50^ however. The problem is that despite recommendations, the majority of knee OA patients do not perform long-term training for various reasons. Due to its time efficiency, joint-friendliness and low subjective effort, WB-EMS training has the potential to reach a large target group of individuals with knee OA who are not receptive to physical training. In the current study, WB-EMS proved to be feasible and well tolerated. The participation rate was high (88%) and only 2 people discontinued the intervention, which indicates good tolerability and high acceptance. Future research should determine under which specific patient characteristics (medical or psychological) WB-EMS might be the better alternative for the treatment of knee OA. For patients with pronounced pain and resulting kinesiophobia, WB-EMS could also be a promising way to reduce pain and build strength, thus creating an ideal basis for conventional strength training.

## Data Availability

Data relative to this work will be available upon reasonable request to the corresponding author.
